# DSTYK predicts Chemoresistance in Triple-Negative Breast Cancer Patient-Derived Xenograft Models

**DOI:** 10.21203/rs.3.rs-9321136/v1

**Published:** 2026-05-07

**Authors:** Samuel Rojas, Jinyu Zhang, Brianna M Elam, Genevieve Schwarz, Yue Zou, Yong Jiang

**Affiliations:** University of Toledo; The University of Toledo College of Medicine and Life Sciences; East Tennessee State University; The University of Toledo College of Medicine and Life Sciences; The University of Toledo College of Medicine and Life Sciences; The University of Toledo College of Medicine and Life Sciences

**Keywords:** Breast cancer, Orthotopic PDX mouse model, PDX expansion, Subcutaneous PDX mouse model, TNBC

## Abstract

Patient-derived xenograft (PDX) models are widely regarded as robust preclinical platforms because they preserve the histopathological and molecular features of primary tumors. In this study, we established twenty triple-negative breast cancer (TNBC) PDX models using freshly resected patient tumors, including ten with high DSTYK expression and ten with low DSTYK expression. Tumor fragments were orthotopically implanted into the fourth mammary fat pads of NSG mice. DSTYK expression was validated by immunohistochemistry and western blotting. Histological evaluation across three serial passages demonstrated that PDX tumors retained the cellular morphology, stromal architecture, and lineage characteristics of their corresponding primary tumors. Using these models, we assessed *in vivo* responses to combination chemotherapy with doxorubicin and docetaxel. DSTYK-low PDX tumors exhibited significantly greater chemosensitivity, whereas DSTYK-high tumors showed marked resistance, indicating a critical role for DSTYK in mediating chemotherapy resistance. Consistent with these findings, analyses from The Human Protein Atlas and Kaplan-Meier Plotter databases revealed that high DSTYK expression is associated with poor survival outcomes in TNBC patients. Collectively, our results from clinical specimens, PDX models, and public datasets identify DSTYK as a promising prognostic biomarker and predictor of chemotherapeutic response in TNBC, with potential implications for patient stratification and treatment optimization.

## Introduction

Triple-negative breast cancer (TNBC) is characterized by the absence of estrogen receptor (ER), progesterone receptor (PR), and human epidermal growth factor receptor 2 (HER2) expression^1^. TNBC represents the most aggressive subtype of breast cancer, with high rates of recurrence, pronounced molecular heterogeneity, and limited targeted therapeutic options^2,3^. Currently, chemotherapy remains the primary neoadjuvant treatment for TNBC. However, the frequent development of chemoresistance is a major contributor to treatment failure and poor clinical outcomes^4–6^. Despite extensive investigation, the molecular mechanisms underlying chemoresistance in TNBC remain incompletely understood, underscoring an urgent need to identify novel biomarkers and therapeutic targets to improve patient survival^7,8^.

Among commonly used regimens, the combination of doxorubicin (DOX) and docetaxel (DXL) has demonstrated substantial efficacy in breast cancer and has been widely applied in clinical trials for TNBC treatment^9–11^. A deeper understanding of how chemotherapy-induced cell death is regulated will provide new strategies to overcome chemoresistance in TNBC^12,13^. Cancer progression is fundamentally driven by an imbalance between cell proliferation and programmed cell death, particularly apoptosis^14,15^. Under normal physiological conditions, apoptosis serves as a critical safeguard to eliminate damaged or transformed cells; however, cancer cells frequently evade apoptosis by inactivating key regulatory pathways^16^. Consequently, successful chemotherapeutic agents exert their antitumor effects largely by reactivating apoptotic signaling in cancer cells^17–19^. In our previous studies, we demonstrated that dual serine/threonine and tyrosine protein kinase (DSTYK) suppresses chemotherapy-induced apoptosis in TNBC cells⁷. Building upon these findings, the present study aims to further elucidate the role of DSTYK in regulating apoptosis and chemotherapeutic response using PDX models.

Recently, trophoblast cell-surface antigen 2 (Trop-2) has emerged as an important biomarker for TNBC ^20–22^. Trop-2, encoded by the TACSTD2 gene, is overexpressed in multiple malignancies and strongly associated with chemoresistance in lung, pancreatic, colorectal, hepatic cancers, and TNBC^11–15^. Functionally, Trop-2 promotes tumor cell survival, proliferation, and metastasis^23^. Notably, Trop-2-targeting antibody–drug conjugates have shown remarkable clinical efficacy, significantly improving progression-free and overall survival in patients with metastatic TNBC^24^. In this study, we observed a positive correlation between DSTYK and Trop-2 expression in clinical TNBC specimens, suggesting a potential functional link between these two molecules.

Previous work from our group and others has shown that DSTYK promotes transforming growth factor-β (TGF-β)–induced epithelial-to-mesenchymal transition (EMT) in colorectal cancer cells^16^ and enhances chemoresistance in TNBC using cellular and orthotopic mouse models^7^. Mechanistically, DSTYK activates ERK signaling^17,18^, downregulates the pro-apoptotic protein Bax^7^, promotes STING pathway activation^19^, and inhibits apoptosis induced by UV irradiation in skin cells^20^. In addition, inhibition of DSTYK sensitizes cancer cells to taxane-based chemotherapy and enhances T cell–mediated cytotoxicity by suppressing autophagy-dependent survival pathways^21^. However, these studies have largely relied on in vitro systems or conventional xenograft models. The role of DSTYK in chemoresistance has not yet been investigated in TNBC PDX models.

PDX models are widely recognized as superior preclinical platforms because they can faithfully recapitulate the histological architecture, molecular heterogeneity, and tumor microenvironment of primary human tumors^2,25,26^. In the present study, we employed DSTYK-high and DSTYK-low TNBC PDX models to evaluate responses to combined DOX/DXL chemotherapy. We demonstrate that DSTYK-high PDX tumors exhibit elevated Trop-2 expression and profound resistance to chemotherapy, whereas DSTYK-low tumors display reduced Trop-2 levels and significantly enhanced chemosensitivity. Collectively, our findings establish DSTYK as a critical determinant of chemotherapeutic response in TNBC and highlight its potential as a prognostic biomarker and therapeutic target for overcoming chemoresistance.

## Materials and Methods

### Establish PDX mouse model and chemotherapeutic assays

All animal experiments were conducted in accordance with protocols approved by the Institutional Animal Care and Use Committee (IACUC) at The University of Toledo (UT). De-identified, fresh, treatment-naïve triple-negative breast cancer (TNBC) specimens were obtained from TNBC patients for the establishment of patient-derived xenograft (PDX) models. Based on DSTYK expression levels, the top ten TNBC specimens with relatively high DSTYK expression and the bottom ten specimens with relatively low DSTYK expression were selected for chemoresistance analyses using PDX models.

NOD-SCID-IL2Rγ^null^ (NSG) mice were housed and maintained in the UT animal facilities. Fresh tumor specimens were sectioned into small fragments (~ 3–5 mm^3^) and aseptically implanted into the fourth mammary fat pad of 6–7-week-old female NSG mice, with one tumor fragment implanted per mouse). Tumor engraftment and growth were monitored regularly. Successfully engrafted tumors were serially passaged to generate P0, P1 (first passage), and P2 (second passage) PDX tumors via subcutaneous expansion. To minimize inter-experimental variability, only tumors from the same generation (P2) were used for all treatment studies.

Once P2 tumors reached an approximate volume of 50 mm^3^, mice were randomized into treatment groups and administered a combination of doxorubicin (DOX) and docetaxel (DXL) via intraperitoneal injection. Treatment was initiated at doses of 4.0 mg/kg DOX and 1.0 mg/kg DXL, and delivered twice weekly for 3–4 weeks. Tumor growth and treatment responses were monitored throughout the treatment period.

### Immunohistochemistry (IHC) Staining

Tumor specimens derived from patients were collected and processed according to our previously described immunohistochemistry protocol^27^. Paraffin-embedded tissue sections (5 μm thick) were incubated overnight at 4°C with the following primary antibodies: anti-DSTYK (OriGene Technologies), anti-Trop2 (Abcam), anti-Ki67 (Abcam), anti-Ku80 (R&D System), anti-CD45, and anti-Bax (Cell Signaling Technology). Immunoreactive signals were visualized using the VECTASTAIN^®^ Elite ABC Kit (Vector Laboratories, Burlingame, CA) in accordance with the manufacturer’s instructions. Hematoxylin was used for nuclear counterstaining. Quantitative analysis of IHC staining intensity was performed using ImageJ software.

### Western Blotting

Western blot (WB) analyses were performed as previously described^27,28^. Briefly, total proteins were extracted from whole-cell lysates, and equal amounts of protein were separated by electrophoresis on 8–10% SDS–polyacrylamide gels. Proteins were then transferred onto polyvinylidene difluoride (PVDF) membranes. The membranes were blocked with 5% non-fat milk and incubated with primary antibodies against Trop2, Bax, DSTYK, and GAPDH (Santa Cruz Biotechnology). After incubation with horseradish peroxidase–conjugated secondary antibodies (Thermo Fisher Scientific), protein signals were detected using enhanced chemiluminescence (ECL; Bio-Rad) and visualized by chemiluminescence imaging. Each Western blot experiment was performed at least three times independently.

### Terminal deoxynucleotidyl transferase-mediated dUTP nick end-labeling (TUNEL) assay

The TUNEL assay was performed as previously described^27,28^, following the manufacturer’s instructions using an apoptosis detection kit (ABP Biosciences, catalog no. A050). Briefly, tissue sections were fixed in 4% paraformaldehyde for 10 min and subsequently washed with phosphate-buffered saline (PBS). Sections were permeabilized with proteinase K (20 μg/mL in PBS). The terminal deoxynucleotidyl transferase (TdT) reaction mixture was freshly prepared and incubated with the sections for 1 h at 37°C in a humidified chamber. Afterward, the slides were counterstained with 4′,6-diamidino-2-phenylindole (DAPI) for 5 min. Fluorescence signals were examined and images were captured using a Leica fluorescence microscope.

### RNA isolation and quantitative real-time PCR

Total RNA was extracted from the cell pellets by the RNeasy Mini kit (Cat# 74104; QIAGEN), and 1 μg RNA was converted to cDNA using the Applied Biosystems High-Capacity cDNA Reverse Transcription Kit (Cat# 43–688-14; Thermo Fisher). Quantitative real-time PCR (real-time-qPCR) was performed using iTaq Universal SYBR Green Supermix (Cat# 1725124, Bio-Rad). DSTYK Forward, 5′-ATTGCCAACCGAAAGCAGGAGG-3′, Reverse, 5′-GGTGCCTACTGGTTCTCCATTC-3′; β-actin Forward 5′-CACCAACTGGGACGACAT-3′, Reverse 5′-ACAGCCTGGATAGCAACG-3′. The gene expression levels were determined by the 2^−ΔΔ^*C*q method and values were normalized to β-actin expression as an internal control.

### Statistical Analysis

Data were analyzed using Prism 7 and are expressed as the mean ± standard deviation (SD). Comparisons among multiple groups were performed using one-way analysis of variance (ANOVA) followed by Tukey’s honestly significant difference post hoc test, with a confidence level of 95%. Comparisons between two groups were made using a parametric paired t-test for normally distributed data or non-parametric Wilcoxon paired t-test for non-normally distributed data. Unless otherwise indicated, experiments included more than five mice or samples per group (*n* > 5). Data were analyzed using the unpaired student t-test, and differences were considered statistically significant at P-values of < 0.05 (*), < 0.01 (**), < 0.001 (***) and < 0.0001 (****) that were considered statistically significant or very significant, respectively.

## Results

### Determination of DSTYK expression levels in human TNBC tissues

**Figure 1:**
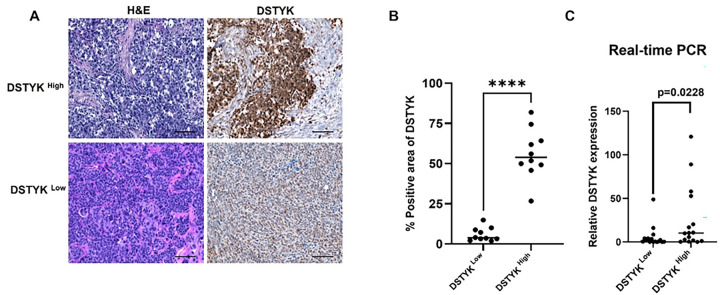
Assessment of DSTYK expression in patient-derived TNBC tumors. (**A**) Representative H&E staining showing the histological morphology of DSTYK-high and DSTYK-low TNBC tumors. Immunohistochemical (IHC) staining specific for DSTYK demonstrates differential protein expression in TNBC tumor samples. Scale bar: 100 mm. (**B**) Quantitative real-time PCR (RT–qPCR) analysis of DSTYK mRNA expression in tumor specimens from DSTYK-high and DSTYK-low TNBC patients. Data are presented as mean ± SD. *P < 0.05; **P < 0.01; ***P < 0.001.

First, histopathological analyses of human triple-negative breast cancer (TNBC) tissues were performed using hematoxylin and eosin (H&E) staining to confirm the characteristic features of breast cancer (Fig. 1A, **left panel**). We then assessed DSTYK protein expression in TNBC tissues by immunohistochemical (IHC) staining (Fig. 1A, **right panel**). Based on IHC staining intensity, we selected the top 10 TNBC specimens with the high DSTYK expression (DSTYK^High^) and the bottom 10 specimens with the low DSTYK expression (DSTYK^Low^). Quantitative analysis was conducted using ImageJ software (Fig. 1B). Consistently, DSTYK mRNA expression was significantly higher in DSTYK^High^ tissues compared with DSTYK^Low^ tissues (Fig. 1C). Together, these results validate the classification of DSTYK^High^ and DSTYK^Low^ TNBC patient specimens.

### DSTYK expression correlates with the expression of cancer stem cell (CSC) markers

**Figure 2:**
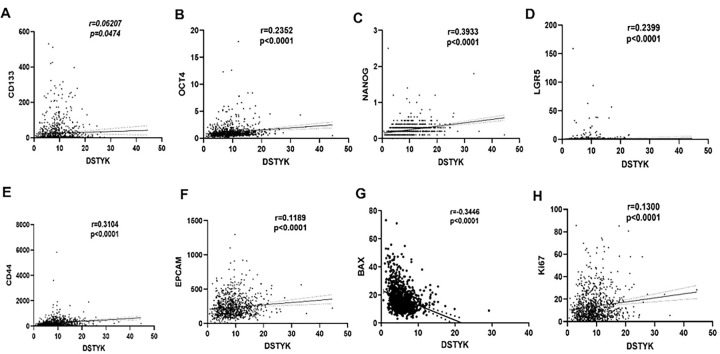
Correlation between DSTYK expression and cancer stem cell-related markers in breast cancer based on data from The Human Protein Atlas (HPA). (**A**) Correlation between DSTYK and CD133. (**B**) Correlation between DSTYK and OCT4. (**C**) Correlation between DSTYK and NANOG. (**D**) Correlation between DSTYK and LGR5. (**E**) Correlation between DSTYK and CD44. (**F**) Correlation between DSTYK and EPCAM. (**G**) Correlation between DSTYK and Ki67. (**H**) Correlation between DSTYK and BAX. Data are presented as mean ± standard deviation (S.D.). Statistical significance is indicated as the P value. P < 0.05 means a significant difference.

The association between DSTYK expression and breast cancer stem cell (CSC) markers was systematically evaluated based on The Human Atlas database. DSTYK expression showed a modest but significant positive correlation with CD133 (r = 0.0621, p = 0.0474; Fig. 2A). Further analyses revealed that DSTYK was positively correlated with multiple established CSC markers, including OCT4 (r = 0.2352, p < 0.0001; Fig. 2B), NANOG (r = 0.3933, p < 0.001; Fig. 2C), LGR5 (r = 0.2399, p < 0.0001; Fig. 2D), CD44 (r = 0.3104, p < 0.0001; Fig. 2E), and EPCAM (r = 0.1189, p < 0.0001; Fig. 2F).

In addition, DSTYK expression was negatively correlated with the pro-apoptotic marker Bax (r = − 0.3446, p < 0.0001; Fig. 2G), while a positive association was observed between DSTYK and the proliferation marker Ki67 (r = 0.1300, p < 0.0001; Fig. 2H).

Collectively, these findings suggest that elevated DSTYK expression is associated with enhanced cancer stemness, reduced apoptotic potential, and increased proliferative capacity, indicating that DSTYK may contribute to apoptosis inhibition and decreased chemosensitivity in breast cancer cells.

### Identification of DSTYK as a Potential Prognostic Biomarker in TNBC

**Figure 3:**
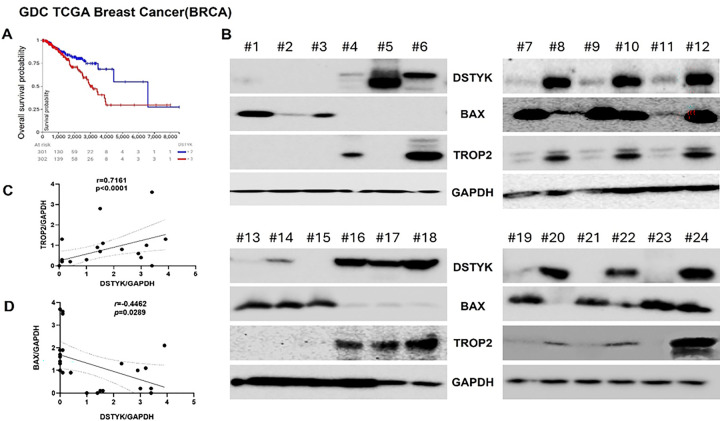
Updated statistical analysis of DSTYK-associated survival probability in breast cancer patients. (**A**) Survival curves comparing overall survival probability in breast cancer patients with high versus low DSTYK expression, based on data from the GDC TCGA Breast Cancer (BRCA) cohort. (**B**) Western blot analysis of DSTYK protein levels in human triple-negative breast cancer (TNBC) tumor tissues, categorized into DSTYK^high^ and DSTYK^low^ groups. (**C**) Correlation analysis between DSTYK and Trop2 protein expressions in breast cancer patient samples. (**D**) Correlation analysis between DSTYK and Bax protein expression in breast cancer patient samples.

To assess the clinical relevance of DSTYK in breast cancer, we analyzed patient data from the GDC TCGA Breast Cancer (BRCA) cohort. Our statistical analysis revealed that elevated DSTYK expression is significantly associated with an increased risk of mortality in breast cancer patients (Fig. 3A), indicating that DSTYK may serve as a negative prognostic factor. These findings further imply a potential role for DSTYK in promoting chemoresistance in breast cancer, including triple-negative breast cancer (TNBC).

Given that Trop2 is highly expressed on the surface of most TNBC cells^21,29^, we next examined the relationship between DSTYK and Trop2 expression. Immunoblotting analyses were performed using randomly selected human TNBC tissue specimens. The results revealed a positive correlation between DSTYK and Trop2 protein levels (Fig. 3B). Quantification of immunoblot band intensities using ImageJ, followed by statistical analysis, confirmed a significant positive correlation between DSTYK and Trop2 expression (Fig. 3C). Based on these immunoblotting results, TNBC tissues were stratified into relative DSTYK^High^ and DSTYK^Low^ groups. Subsequent statistical analysis demonstrated that the pro-apoptotic factor Bax was negatively correlated with DSTYK expression (Fig. 3B & 3D). Collectively, these data suggest that DSTYK may function as a tumor-promoting element in TNBC by modulating the expression of Trop2 and Bax, thereby contributing to tumor progression and resistance to chemotherapeutic drug-induced apoptosis.

### Establishment of TNBC PDX Models in NSG Mice

**Figure 4:**
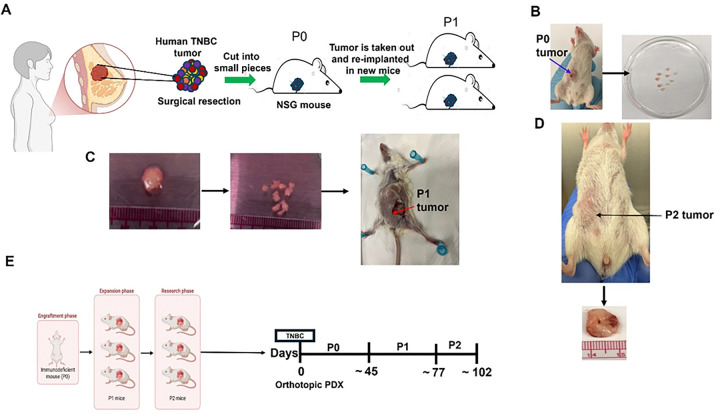
Establishment of patient-derived xenograft (PDX) mouse models. (**A**) NOD-SCID-IL2Rγ^null^ (NSG) mice (The Jackson Laboratory) were used for tumor implantation. Fresh patient tumor specimens were cut into small fragments (~5 mm^3^) and aseptically implanted into the fourth mammary fat pad of 6–7-week-old female mice (one fragment per mouse) to generate P0 tumors. (**B**) P0 tumors were harvested, sectioned into ~5 mm^3^ fragments, and used for subsequent transplantation. (**C**) Tumor fragments from P0 tumors were aseptically implanted into the fourth mammary fat pad of 6–7-week-old female mice (one fragment per mouse) to generate P1 tumors; the same procedure was repeated to generate P2 tumors. (**D**) Representative P2 tumors derived from P1 tumors. (**E**) Schematic timeline and workflow of PDX model establishment.

To establish PDX models, NOD-SCID-IL2Rγ^null^ (NSG) mice were used as illustrated in Fig. 4. For each tumor specimen, ten mice were employed. Fresh TNBC tumor tissues were aseptically sectioned into small fragments (~ 5 mm^3^) and orthotopically implanted into the fourth mammary fat pad of 6–7-week-old female NSG mice, with one tumor fragment implanted per mouse (Fig. 4).

The first passage (P0) of subcutaneous TNBC PDX tumors required approximately 6–8 weeks for expansion, whereas the second (P1) and third (P2) passages required approximately 4 weeks and 3–4 weeks, respectively. All subcutaneous PDX tumors were expanded to a volume of ~ 300–400 mm^3^ prior to subsequent transplantation.

**Figure 5:**
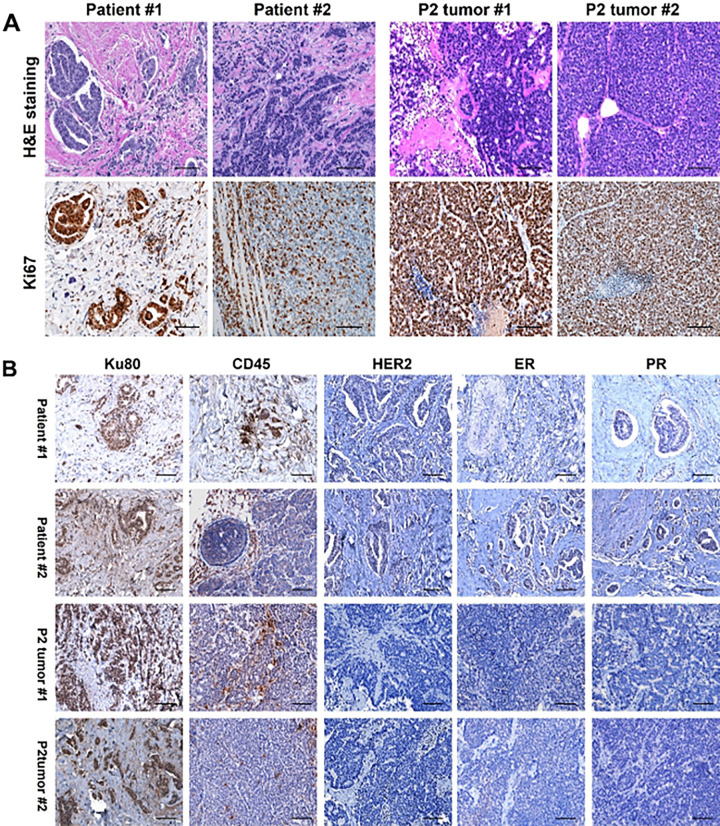
P2 tumors preserve original human tumor features. (**A**) *H&E* staining and Ki67 staining in representative P2 PDX tumors compared to original human TNBC tumors. Scale bar: 100 mm. (**B**) IHCstaining for Ku80 and CD45 was performed to confirm the human origin of the P2 tumors. ER, PR, and HER2 staining further verified the maintenance of the triple-negative phenotype.

Following orthotopic implantation, P1 and P2 PDX tumors demonstrated significantly accelerated in vivo growth compared with P0 tumors, with growth rates significantly increased (Fig. 4E). To assess whether key characteristics of the original tumors were preserved during passaging, randomly selected PDX models derived from two patients with triple-negative breast cancer (TNBC) were subjected to histopathological evaluation. Hematoxylin and eosin (H&E) staining and Ki67 immunohistochemistry of P2 tumors confirmed retention of original TNBC histological features and high proliferative activity (Fig. 5A). Immunohistochemical analysis of estrogen receptor (ER), progesterone receptor (PR), and HER2 further verified maintenance of the triple-negative phenotype (Fig. 5B). Moreover, Ku80 and CD45 staining confirmed the human origin of the tumor cells and revealed minimal murine hematopoietic cell contamination, supporting the fidelity of the PDX models (Fig. 5B).

### DSTYK expression correlates with Trop2 upregulation and Bax suppression in clinical TNBC.

Our previous studies demonstrated that chemoresistant TNBC cells regain chemosensitivity upon DSTYK knockout (KO) following chemotherapeutic treatment^9^. To explore the mechanisms by which DSTYK promotes chemoresistance in TNBC, we performed immunohistochemical (IHC) analyses to evaluate the expression of DSTYK, Trop2, and Bax in paired primary TNBC specimens and corresponding residual tumors obtained after neoadjuvant chemotherapy.

**Figure 6:**
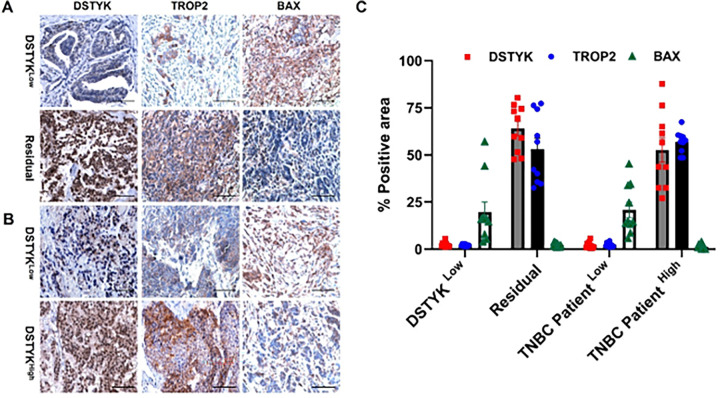
DSTYK serves as a prognostic biomarker in clinical TNBC. (**A**) Representative IHC staining images showing the expression of DSTYK, Trop2, and Bax in DSTYK^Low^ TNBC tissues and the corresponding residual TNBC tissues. (**B**) Representative IHC images comparing the expression levels of DSTYK, Trop2, and Bax in TNBC tissues with high versus low DSTYK expression. (**C**) Quantitative analysis of IHC staining from panels (A) and (B).

IHC staining revealed that DSTYK protein expression was significantly increased in residual TNBC tissues compared with their matched pre-treatment DSTYK^Low^ tumors (Fig. 6A, B). Consistently, Trop2 expression was markedly upregulated in residual tumors following chemotherapy. In contrast, expression of the pro-apoptotic protein Bax was significantly reduced in residual TNBC tissues relative to untreated DSTYK^Low^ tumors (Fig. 6A, C).

Correlation analyses further demonstrated a positive association between DSTYK and Trop2 expression and an inverse relationship between DSTYK and Bax expression. Specifically, TNBC tissues with low DSTYK expression exhibited reduced Trop2 levels and elevated Bax expression, whereas tumors with high DSTYK expression showed increased Trop2 expression and concomitant suppression of Bax (Fig. 6B, C).

Collectively, these findings suggest that Trop2 and Bax may serve as downstream effectors of DSTYK signaling. Our data identify Trop2 and Bax as novel components of a DSTYK-associated regulatory network that contributes to chemoresistance in TNBC and supports DSTYK as a potential prognostic biomarker in the clinical setting.

### DSTYK Promotes Chemoresistance in In Vivo PDX Models

**Figure 7:**
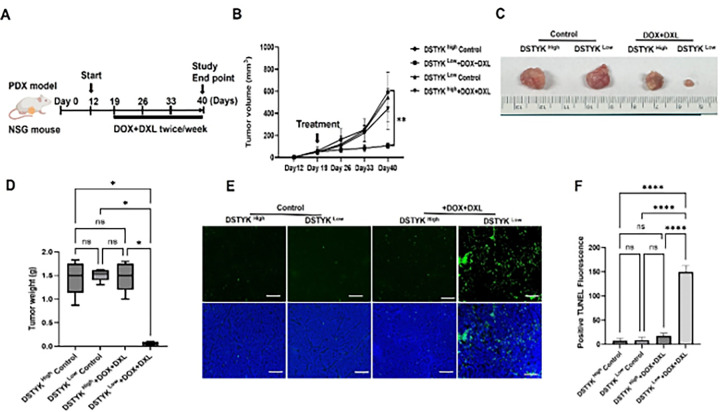
Reduced DSTYK expression enhances chemosensitivity in PDX models. (**A**) Schematic illustration of the treatment regimen used in PDX mouse models receiving combined doxorubicin (DOX) and docetaxel (DXL) therapy. (**B**) Tumor growth curves of PDX models stratified by DSTYK expression (DSTYK^High^ and DSTYK^Low^) with or without drug treatment, as indicated. Data are presented as mean ± S.D. (**C**) Representative images of excised tumors from DSTYK^High^ and DSTYK^Low^ PDX groups with and without drug treatment. (**D**) Quantification of tumor weights at the experimental endpoint. Data are shown as mean ± S.D. (**E**) TUNEL assays assessing cell death in tumor tissues from DSTYK^High^ and DSTYK^Low^ groups with and without drug treatment. (**F**) Quantification of TUNEL-positive cells from panel (E). Data are presented as mean ± S.D. Statistical significance is indicated as follows: *P < 0.05; **P < 0.01; ***P < 0.001; ****P < 0.0001.

To provide clinically relevant evidence that DSTYK contributes to chemoresistance, we employed patient-derived xenograft (PDX) mouse models. The experiment procedure is depicted in Fig. 7A.

Following orthotopic implantation, tumor volume, tumor weight, and body weight were monitored twice a week, and tumor growth curves were generated to compare therapeutic responses between tumors with high versus low DSTYK expression, no significant differences in tumor growth were observed between DSTYK^high^ and DSTYK^low^ PDX tumors (Fig. 7B), consistent with our previous findings that DSTYK does not significantly influence cancer cell proliferation^28^. In contrast, after DOX + DXL treatment, tumors derived from the DSTYK^low^ group exhibited marked regression compared with those from the DSTYK^high^ group (Fig. 7B–D).

Consistently, TUNEL staining revealed a significantly higher level of apoptosis in DSTYK^Low^ tumors following chemotherapy compared with DSTYK^High^ tumors (Fig. 7E & F). These findings indicate that elevated DSTYK expression attenuates chemosensitivity by suppressing chemotherapy-induced tumor cell death.

Taken together, these results demonstrate that DSTYK plays a pivotal role in promoting chemoresistance in TNBC. Targeting DSTYK in combination with standard chemotherapy may therefore represent a promising strategy to enhance therapeutic efficacy in TNBC patients.

## Discussion

This study demonstrates that primary TNBC tumors with elevated DSTYK expression exhibit significantly greater chemoresistance than those with lower DSTYK levels. DSTYK expression is positively correlated with the newly identified TNBC marker Trop2 and negatively correlated with the pro-apoptotic protein Bax. Together with DSTYK and Trop2, Bax may therefore also serve as a potential biomarker for assessing chemoresistance in TNBC, consistent with several previous reports^18,19,30^. Furthermore, in our PDX mouse models, tumors characterized by high DSTYK and low Bax expression displayed markedly reduced sensitivity to drug treatment compared with tumors expressing lower DSTYK and higher Bax levels.

Our previous studies revealed that DSTYK suppresses chemotherapy-induced apoptosis in TNBC cells and destabilizes Bax in colorectal cancer cells^27^. In the present study, we extend these findings by providing clinical evidence that DSTYK and Bax protein levels are inversely correlated in TNBC specimens. Combined with our functional PDX data showing that DSTYK inhibits apoptosis and attenuates chemosensitivity, these results strongly suggest that DSTYK may similarly downregulate Bax stability in TNBC. Previously, we demonstrated that chemoresistant cancer cells regain chemosensitivity following DSTYK knockout^28^, here in, immunohistochemical analysis of clinical TNBC samples reveals that DSTYK protein levels are significantly elevated in residual tumors after neoadjuvant chemotherapy compared with treatment-naïve tumors. This observation suggests that DSTYK may be a key determinant driving the chemoresistant phenotype of residual TNBC. Collectively, these findings reinforce our earlier discovery and establish DSTYK as a potential prognostic biomarker and therapeutic target for chemoresistant TNBC.

To further characterize the role of DSTYK in chemoresistance, we employed patient-derived xenograft (PDX) mouse models generated from clinical TNBC specimens. PDX models are highly valued in cancer research^12,25,31^, particularly for TNBC, as they better mirror the characteristics of human disease compared with conventional cancer cell lines^32^. By engrafting freshly resected patient tumor tissues into immunodeficient mice, PDX models preserve tumor architecture, histopathological features, and intratumoral heterogeneity, and these factors that are critical for accurately assessing therapeutic response. In our PDX studies, tumors with low DSTYK expression exhibited significantly greater regression following chemotherapy than those with high DSTYK expression. These findings further support the conclusion that elevated DSTYK levels confer increased chemoresistance. To enhance the translational relevance of our work, future studies should evaluate the impact of DSTYK on resistance to additional chemotherapeutic agents beyond doxorubicin and docetaxel.

Statistical analysis of the patient dataset indicates that a high DSTYK level is correlated with an increased risk of mortality in breast cancer patients, TNBC patients, and TNBC patients treated only with chemotherapy^28^. However, the molecular mechanisms by which DSTYK mediates chemoresistance remain poorly understood^33^. Notably, we identified a strong positive correlation between DSTYK and Trop2 expression. Trop2 is a well-established therapeutic target in TNBC, as it is highly expressed on the surface of most TNBC cells while exhibiting minimal expression in normal tissues. Trop2 is a transmembrane glycoprotein that functions as an intracellular calcium signal transducer and plays a critical role in tumor progression by regulating oncogenic signaling pathways, including AKT, ERK, and JAK/STAT. Trop2 overexpression is consistently associated with poor prognosis and aggressive TNBC phenotypes^32,34,35^. Trop2 antibody drug conjugates sacituzumab govitecan (Trodelvy) and datopotomab deruxtecan (Datroway) have been approved by FDA not long ago for the clinical treatment of TNBC patients, which is a groundbreaking achievement to significantly increase both progression-free survival (PFS) and overall survival (OS) probability of metastatic TNBC patients according to a statistical analysis^24,36^. In our study, TNBC specimens with high DSTYK expression consistently exhibited elevated Trop2 levels, whereas tumors with low DSTYK expression showed correspondingly low Trop2 expression. This statistically significant correlation suggests a previously unrecognized link between DSTYK and Trop2 and highlights a compelling direction for future mechanistic studies aimed at elucidating how DSTYK promotes chemoresistance. Given that DSTYK is a protein kinase, it represents a highly druggable target. The development of cell-permeable DSTYK inhibitors may therefore offer a novel therapeutic strategy to overcome chemoresistance and enhance the efficacy of existing treatments, including Trop2-targeted therapies, ultimately improving clinical outcomes for patients with TNBC.

## Conclusion

In summary, our findings provide important advances in therapeutic strategies for the ongoing challenge of chemotherapy resistance in TNBC. By further validating, we identify DSTYK as a promising druggable target for targeting and eliminating chemoresistant TNBC in clinical patients.

## Supplementary Material

Supplementary Files

This is a list of supplementary files associated with this preprint. Click to download.

• S1.jpg

• S2.jpg

• S3.jpg

• S4.jpg
